# Third generation cognitive-behavioral therapies for major depressive disorder- a literature review

**DOI:** 10.1192/j.eurpsy.2021.873

**Published:** 2021-08-13

**Authors:** D. Vasile, O. Vasiliu, D. Vasiliu

**Affiliations:** Psychiatry, University Emergency Central Military Hospital Dr. Carol Davila, Bucharest, Romania

**Keywords:** cognitive-behavioral therapy, major depressive disorder, mindfulness

## Abstract

**Introduction:**

Cognitive behavioral therapies (CBT) represent a heterogeneous group of psychotherapies in continuous development that share a directive, structured, collaborative approach. Due to a high degree of treatment-resistant cases of major depressive disorder (MDD), new augmentation therapies are urgently needed, in order to increase the chance of recovery in these patients.

**Objectives:**

To analyze data that may support the indication of third wave CBT in patients with MDD.

**Methods:**

A literature search was performed in the main electronic databases, and papers published between January 2000 and August 2020 were included.

**Results:**

Acceptance and commitment therapy has been associated with positive results, but data are derived from low quality trials (n=2). Dialectical-behavioral therapy (DBT)-based skill group have been also associated with favorable outcome, in MDD patients (n=2). Mindfulness-based cognitive therapy (MBCT) was also proven effective in the treatment in MDD (n=4), treatment-resistant MDD included, but the difference between MBCT and active comparators was not always significant. Metacognitive therapy (MCT) has been evaluated in good quality clinical trials (n=4), and its efficacy was confirmed. Mild and moderate MDD patients may benefit from compassion-focused therapy (CFT) (n=1). Behavioral activation (BA) is dedicated to MDD patients and according to a meta-analysis (n=26 randomized controlled trials) BA is superior to other active comparators, although the quality of clinical trials was modest.

**Conclusions:**

Third generation CBT could be useful in MDD patients as augmentative strategy, but more good-quality data are necessary before recommending them in an evidence-based treatment guideline as a distinctive intervention from classical CBT.

